# Distinct Gut Microbiome Profiles Underlying Cardiometabolic Risk Phenotypes in Individuals with Obesity

**DOI:** 10.3390/nu18020353

**Published:** 2026-01-22

**Authors:** Iveta Nedeva, Yavor Assyov, Veselka Duleva, Vera Karamfilova, Zdravko Kamenov, Julian Naydenov, Teodora Handjieva-Darlenska, Venelin Denchev, Alexander Kolevski, Victoria Pencheva, Vlayko Vodenicharov

**Affiliations:** 1 Department of Epidemiology and Hygiene, Medical University Sofia, 1431 Sofia, Bulgaria; v.vodenicharov@medfac.mu-sofia.bg; 2 Department of Internal Diseases, Medical University Sofia, 1431 Sofia, Bulgaria; yassyov@medfac.mu-sofia.bg (Y.A.); vkaramfilova@medfac.mu-sofia.bg (V.K.); zkamenov@medfac.mu-sofia.bg (Z.K.); julij.n@abv.bg (J.N.); 3 National Center of Public Health and Analyses, 1431 Sofia, Bulgaria; 4 Department of Pharmacology and Toxicology, Medical University Sofia, 1431 Sofia, Bulgaria; tdarlenska@medfac.mu-sofia.bg (T.H.-D.); v.denchev@medfac.mu-sofia.bg (V.D.); 5 Department of Microbiology, University Hospital “Alexandrovska”, 1431 Sofia, Bulgaria; al.kolevski@gmail.com (A.K.); penchevvictoria@gmail.com (V.P.)

**Keywords:** gut microbiome, obesity, metabolic syndrome, cardiometabolic risk

## Abstract

**Background:** Obesity-related cardiometabolic disorders have been linked to alterations in selected gut microbiome components, yet clinically relevant microbial signatures remain incompletely defined. **Objectives**: This study investigated associations between selected gut bacterial taxa and cardiometabolic risk phenotypes in individuals with obesity. **Methods**: In this cross-sectional study, 100 adults with obesity were stratified according to metabolic syndrome status. Gut microbiome composition was assessed using targeted multiplex real-time PCR of functionally relevant bacterial taxa. Associations with anthropometric and cardiometabolic parameters were examined using correlation analysis, ROC curves, and multivariable logistic regression models. **Results**: Reduced relative abundance of *Lachnospiraceae* was associated with metabolic syndrome, lower *Faecalibacterium* abundance with arterial hypertension, and increased *Prevotella* abundance with dyslipidemia. ROC analyses identified cohort-specific discriminative thresholds with moderate accuracy. **Conclusions**: Selected taxon-specific gut microbiome signatures are associated with cardiometabolic risk phenotypes in obesity. These findings are exploratory and require validation in longitudinal and independent cohorts.

## 1. Introduction

Obesity represents a central component of metabolic syndrome and is strongly associated with increased risk of type 2 diabetes mellitus and cardiovascular disease [1,2]. At the physiological level, obesity develops as a consequence of chronic positive energy balance, leading to excessive expansion and functional remodeling of adipose tissue [3]. This process promotes adipose tissue inflammation, altered adipokine secretion, and systemic metabolic dysfunction, which collectively underpin the development of metabolic syndrome [4].

The gut microbiome, defined as the collective genomes of trillions of microorganisms inhabiting the human gastrointestinal tract, has emerged as a pivotal regulator of host metabolic homeostasis [2,4]. Advances in metagenomics, metabolomics, and systems biology have demonstrated that the gut microbiome exerts profound effects on host energy balance, glucose and lipid metabolism, immune function, and inflammatory signaling. Alterations in gut microbiome composition and functionality, commonly referred to as dysbiosis, have been consistently associated with obesity, insulin resistance, and the development of metabolic syndrome.

In recent years, the gut microbiome has emerged as a critical environmental factor contributing to the development of obesity and metabolic syndrome, providing a mechanistic link between diet, host metabolism, and metabolic disease risk [5,6,7,8,9,10]. The gut microbiome—comprising trillions of microorganisms inhabiting the human gastrointestinal tract—plays a central role in regulating nutrient digestion, energy harvest, lipid and glucose metabolism, bile acid signaling, and immune homeostasis [8,9]. Accumulating evidence indicates that alterations in gut microbiome composition and function, commonly referred to as dysbiosis, are closely associated with excess adiposity, insulin resistance, chronic low-grade inflammation, and other key features of metabolic syndrome [7,10,11].

Mechanistically, the gut microbiome influences host energy balance through enhanced extraction of dietary energy and the production of short-chain fatty acids, which act as both metabolic substrates and signaling molecules involved in appetite regulation, adipogenesis, and insulin sensitivity [12,13]. In addition, dysbiosis-related impairment of gut barrier integrity promotes translocation of bacterial-derived lipopolysaccharides into the circulation, leading to metabolic endotoxemia and activation of pro-inflammatory pathways that exacerbate insulin resistance and adipose tissue dysfunction [14,15]. Alterations in microbiota-driven bile acid metabolism and gut–brain signaling further contribute to metabolic dysregulation by modulating glucose homeostasis, lipid metabolism, and energy expenditure [4,5,16].

Collectively, these findings highlight the gut microbiome as a pivotal mediator in the pathophysiology of obesity and metabolic syndrome and underscore its relevance as a potential target for early diagnosis, prevention, and nutrition-based therapeutic strategies. Despite these advances, gut microbiome alterations associated with early metabolic dysfunction remain incompletely characterized.

Accordingly, the present study aimed to investigate gut microbiome changes in individuals with incipient metabolic disorders and to determine whether distinct microbial signatures discriminate them from metabolically healthy controls, thereby elucidating the role of the gut microbiome in the early pathogenesis of metabolic syndrome.

## 2. Materials and Methods

### 2.1. Study Design

This study was designed as a cross-sectional investigation involving 100 adults with obesity. Participants were stratified into two groups based on metabolic status: individuals with obesity without metabolic syndrome (*n* = 50; Group 1) and individuals with obesity and concomitant metabolic syndrome (*n* = 50; Group 2). The primary objective was to assess gut microbiome composition in both groups and to identify microbial patterns associated with metabolic syndrome. In addition, biochemical parameters and instrumental indicators of metabolic dysfunction were evaluated, and their relationships with gut microbiome profiles were analyzed. Data collection was conducted over a one-year period. This study was conducted in accordance with the principles of the Declaration of Helsinki and was approved by the Ethics Committee of the Medical University of Sofia. Written informed consent was obtained from all participants prior to enrollment.

### 2.2. Eligibility Criteria

#### 2.2.1. Inclusion Criteria

Participants were eligible for inclusion if they met the following criteria:

- Age between 35 and 74 years;
- Body mass index (BMI) ≥ 30 kg/m^2^;
- Presence of prediabetes, defined as fasting plasma glucose levels between 6.1 and 6.9 mmol/L, 2 h plasma glucose values between 7.8 and 11.0 mmol/L during a 75 g oral glucose tolerance test (OGTT), and/or glycated hemoglobin (HbA1c) levels between 5.7% and 6.4%;
- Newly diagnosed type 2 diabetes mellitus, defined as fasting plasma glucose ≥ 7.0 mmol/L, 2 h plasma glucose ≥ 11.1 mmol/L during OGTT, and/or HbA1c ≥ 6.5%, in the absence of antidiabetic therapy.

#### 2.2.2. Exclusion Criteria

Participants were excluded from the study if any of the following conditions were present:

- Evidence of liver dysfunction, defined as serum liver enzyme levels ≥ three times above the upper limit of the reference range;
- Chronic kidney disease stages III–IV;
- Heart failure classified as New York Heart Association (NYHA) functional classes III–IV;
- Active or previous neoplastic disease;
- Use of antibiotics within the previous three months prior to enrollment;
- Use of probiotics, prebiotics, or synbiotics within the previous three months prior to enrollment;
- Current or prior use of metformin or other glucose-lowering medications within the previous three months in order to avoid pharmacological confounding of metabolic and gut microbiome outcomes.

### 2.3. Anthropometric Parameters

Anthropometric measurements included body weight, height, waist circumference, and hip circumference, obtained using standardized procedures. Body mass index (BMI) was calculated as body weight (kg) divided by height squared (m^2^).

Visceral adiposity was assessed using the Visceral Adiposity Index (VAI), calculated according to sex-specific formulas as previously described:

- **For males:**

VAI = [WC/(36.68 + (1.88 × BMI))] × (TG/1.03) × (1.31/HDL)

- **For females:**

VAI = [WC/(36.58 + (1.89 × BMI))] × (TG/0.81) × (1.52/HDL)

where WC is waist circumference (cm), TG represents triglyceride concentration (mmol/L), and HDL denotes high-density lipoprotein cholesterol (mmol/L).

### 2.4. Assessment of Glycemic Homeostasis

Glycemic homeostasis was evaluated using a standard 75 g oral glucose tolerance test (OGTT). Plasma glucose and immunoreactive insulin (IRI) concentrations were measured at baseline (0 min) and 60 and 120 min following glucose ingestion.

Insulin resistance was assessed using the Homeostatic Model Assessment of Insulin Resistance (HOMA-IR), with values > 2.5 considered indicative of insulin resistance.

### 2.5. Definition of Metabolic Syndrome

Metabolic syndrome was defined according to the International Diabetes Federation (IDF) criteria as the presence of central obesity, defined by a waist circumference ≥ 80 cm for women and ≥94 cm for men, in combination with any two of the following metabolic risk factors:

- Fasting plasma glucose ≥ 5.6 mmol/L;
- Blood pressure ≥ 130/85 mmHg or current antihypertensive treatment;
- Triglyceride levels ≥ 1.7 mmol/L;
- HDL cholesterol ≤ 1.03 mmol/L in men and ≤1.29 mmol/L in women.

### 2.6. Assessment of Dietary Habits

Dietary intake was assessed using a combination of a 24 h dietary recall and a qualitative food frequency questionnaire (FFQ) to capture both short-term intake and habitual dietary patterns. The 24 h dietary recall was conducted through structured interviews, during which participants reported all foods and beverages consumed during the previous day, including portion sizes and preparation methods.

### 2.7. Gut Microbiome Analysis by Multiplex Real-Time PCR

Gut microbiome composition was assessed using multiplex real-time polymerase chain reaction (RT-PCR) with commercially available diagnostic kits processed simultaneously according to the manufacturer’s instructions: MutaPLEX^®^ PRE/RU/LA, MutaPLEX^®^ EU/BAC/BIF, and MutaPLEX^®^ AKM/FAEP (Immundiagnostik AG, Bensheim, Germany). One stool sample per participant was collected and analyzed. Samples were not pooled or mixed prior to DNA extraction.

### 2.8. Principle of the Method

The applied kits are designed for the quantitative detection of bacterial DNA from fecal samples using real-time PCR. Each kit contains taxon-specific primers and dual-labeled hydrolysis probes enabling simultaneous amplification and fluorescence-based detection of selected bacterial taxa relevant to metabolic health: *Prevotella* spp., *Ruminococcus* spp., and members of the *Lachnospiraceae* family (MutaPLEX^®^ PRE/RU/LA); *Eubacterium rectale*, *Bacteroides* spp., and *Bifidobacterium* spp. (MutaPLEX^®^ EU/BAC/BIF); and *Akkermansia muciniphila* and *Faecalibacterium prausnitzii* (MutaPLEX^®^ AKM/FAEP). During amplification, probe hydrolysis results in an increase in fluorescence intensity proportional to the amount of target bacterial DNA present.

### 2.9. Procedure

DNA extraction. Microbial DNA was extracted from fecal samples using the MutaCLEAN^®^ Universal RNA/DNA Kit (KG1038) (Immundiagnostik AG, Bensheim, Germany) according to the manufacturer’s protocol. An internal control DNA was added to each sample to monitor extraction efficiency and PCR performance.

Quantitative analysis. Quantification was performed using standard curves generated from three calibration standards of known concentration provided with the kits. Results were initially expressed as DNA copy numbers per reaction. Conversion to bacterial cells per gram of feces was calculated using

Nmicroorganism (cells/g feces) = n copies/reaction × K (1/g)

where K is a correction factor determined by extraction parameters and sample mass. Following absolute quantification, relative abundances (%) were calculated for comparative analyses across study groups.

### 2.10. Statistical Analysis

Statistical analyses were performed using SPSS software, version 25.0 (IBM Corp., Armonk, NY, USA). Data distribution was assessed using the Kolmogorov–Smirnov test. Continuous variables are presented as mean ± standard deviation or median (interquartile range), as appropriate, while categorical variables are expressed as frequencies and percentages.

Comparisons between the two study groups were conducted using Student’s *t*-test for normally distributed variables or the Mann–Whitney *U* test for non-normally distributed variables.

Multivariate logistic regression analysis was performed to evaluate independent associations between gut microbiome parameters and the presence of metabolic syndrome, with results expressed as odds ratios (ORs) and 95% confidence intervals (CIs).

Heatmap visualization was used for exploratory analysis of associations between gut microbiome taxa and metabolic parameters, based on standardized (z-score–transformed) data and hierarchical clustering.

A two-sided *p* value < 0.05 was considered statistically significant.

Sex was included as a covariate in all multivariable logistic regression models to account for potential sex-related differences in cardiometabolic risk and gut microbiome composition.

## 3. Results

The study cohort comprised 100 participants with a mean age of 48.16 ± 10.89 years. The sample included 60 women (60%) and 40 men (40%). Participants were stratified into two groups based on metabolic status: Group 1 consisted of 50 individuals with obesity without metabolic syndrome, while Group 2 included 50 participants with obesity and metabolic syndrome. Baseline demographic, anthropometric, and metabolic characteristics of the two groups are presented in Table 1.

Compared with participants with obesity alone, individuals with obesity and metabolic syndrome exhibited significantly higher waist circumference, WHR, WSR, body fat percentage, and visceral fat rating, while age and BMI did not differ significantly between groups.

As shown in Table 2, participants with obesity and metabolic syndrome exhibited significantly higher systolic blood pressure (*p* = 0.023), total cholesterol (*p* = 0.042), and triglyceride levels (*p* = 0.007), as well as a higher prevalence of hypertension (*p* = 0.006), smoking (*p* < 0.001), and dyslipidemia (*p* = 0.002), compared with individuals with obesity alone.

**Figure 1:**
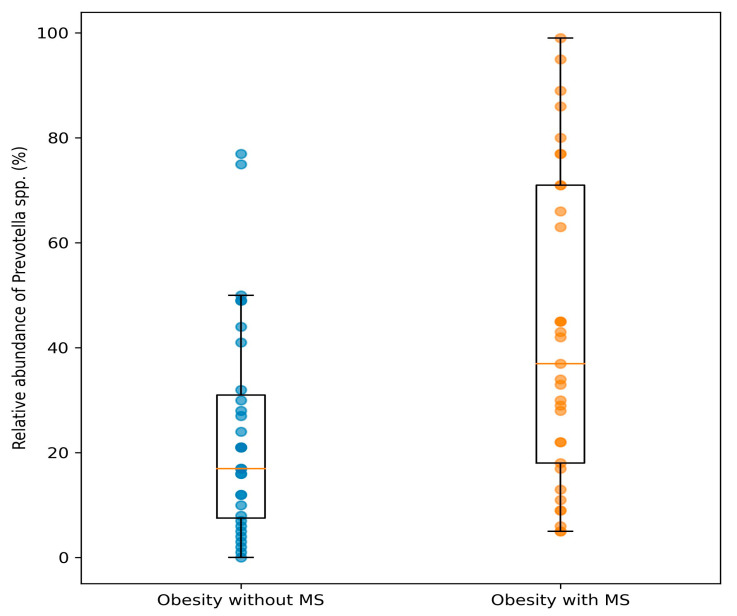
Relative abundance of *Prevotella* spp. (%) in individuals with obesity without and with metabolic syndrome. Data are presented as median and interquartile range, with individual data points illustrating substantial interindividual variability.

Analysis of functionally relevant bacterial groups revealed significant differences in relative abundance. Participants with metabolic syndrome exhibited a significantly higher relative abundance of *Prevotella* spp. compared with those without metabolic syndrome (43.09% ± 29.30% vs. 23.39% ± 20.44%, *p* = 0.004) (Figure 1).

**Figure 2:**
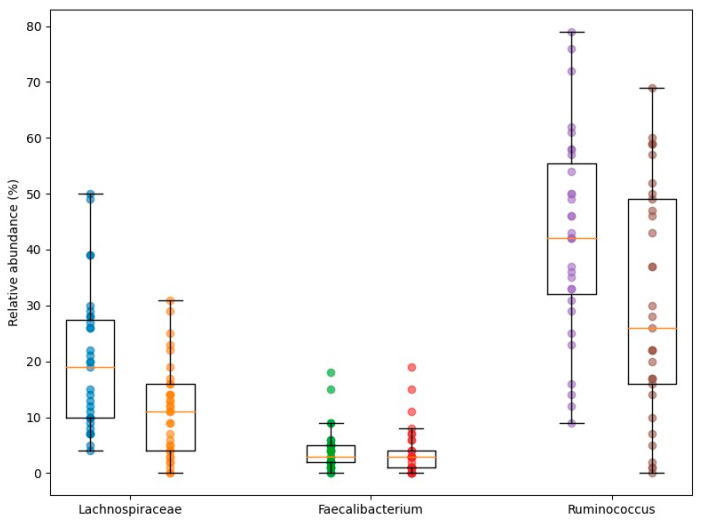
Relative abundance (%) of selected SCFA-producing and functionally relevant gut bacterial taxa (*Lachnospiraceae*, *Faecalibacterium*, and *Ruminococcus*) in individuals with obesity without and with metabolic syndrome. Data are presented as median and interquartile range, with individual data points illustrating substantial interindividual variability.

Conversely, the relative abundance of *Ruminococcus* spp. was significantly lower in the metabolic syndrome group (30.33% ± 20.85%) than in participants without metabolic syndrome (42.58% ± 18.19%, *p* = 0.015). A similar reduction was observed for members of the *Lachnospiraceae* family (11.27% ± 8.33% vs. 19.84% ± 12.52%, *p* = 0.005) (Figure 2).

Microbiome-derived ratio analysis further supported these findings. The *Bacteroides*/*Faecalibacterium* ratio did not differ significantly between groups (1.51 ± 2.05 vs. 2.12 ± 3.25, *p* = 0.995), although wide dispersion of values was observed, indicating pronounced interindividual heterogeneity.

Correlation analyses revealed distinct and taxon-specific associations between gut microbial composition and cardiometabolic parameters. Relative *Bacteroides* abundance (%) showed significant positive associations with fasting insulin (*r* = 0.283, *p* < 0.05), HbA1c (*r* = 0.330, *p* < 0.05), serum uric acid (*r* = 0.261, *p* < 0.05), and LDL cholesterol (*r* = 0.219, *p* < 0.05). With respect to instrumental parameters, higher relative *Bacteroides* abundance was strongly inversely associated with markers of peripheral neuropathy and arterial stiffness, including biothesiometry (*r* = −0.701, *p* < 0.001) and the cardio-ankle vascular index (CAVI; *r* = −0.554, *p* < 0.05).

In contrast, relative *Ruminococcus* abundance (%) was inversely associated with fasting glucose (*r* = −0.256, *p* < 0.05), fasting insulin (*r* = −0.284, *p* < 0.05), gamma-glutamyl transferase (GGT; *r* = −0.398, *p* < 0.01), and triglyceride levels (*r* = −0.319, *p* < 0.05). Relative *Lachnospiraceae* abundance (%) demonstrated inverse associations with age (*r* = −0.308, *p* < 0.05), waist circumference (*r* = −0.283, *p* < 0.05), and body fat percentage (*r* = −0.318, *p* < 0.05). Moreover, significant inverse correlations were observed with fasting glucose (*r* = −0.279, *p* < 0.05), plasma glucose at 0 min during the oral glucose tolerance test (OGTT; *r* = −0.271, *p* < 0.05), and HOMA-IR (*r* = −0.284, *p* < 0.05). Relative *Eubacterium* abundance (%) was positively associated with plasma glucose at 0 min during OGTT (*r* = 0.313, *p* < 0.05), fasting insulin (*r* = 0.434, *p* < 0.05), LDL cholesterol (*r* = 0.294, *p* < 0.05), and total cholesterol (*r* = 0.264, *p* < 0.05), while no significant associations were observed with triglyceride levels or HbA1c. In addition, both absolute and relative *Eubacterium* abundance were positively correlated with arterial stiffness, as assessed by CAVI (*r* = 0.511, *p* < 0.05). Finally, relative *Prevotella* abundance (%) showed significant positive correlations with LDL cholesterol (*r* = 0.363, *p* < 0.05), triglyceride levels (*r* = 0.411, *p* < 0.05), and total cholesterol (*r* = 0.444, *p* < 0.05).

**Figure 3:**
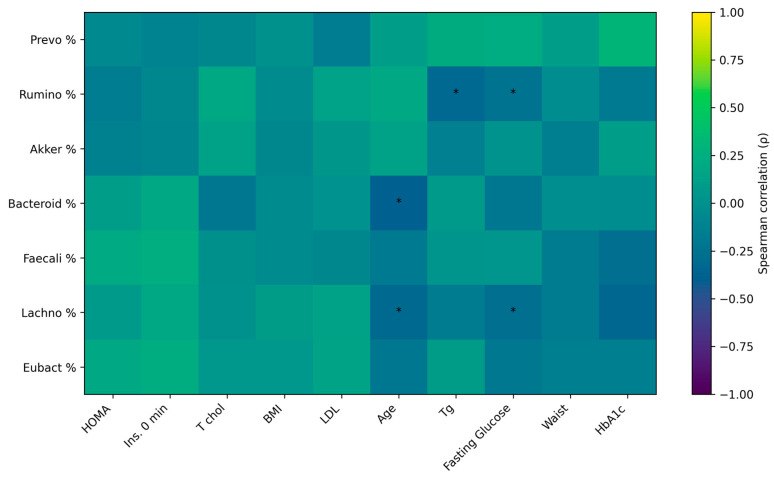
xHeatmap of Spearman correlation coefficients (ρ) illustrating associations between selected gut bacterial taxa (relative abundance, %) and anthropometric and cardiometabolic parameters. Asterisks indicate statistically significant correlations (* *p* < 0.05). The heatmap reflects associations within a predefined set of functionally relevant taxa quantified by targeted RT-qPCR and does not represent comprehensive gut microbiome profiling.

Consistent with the correlation analyses, heatmap visualization of standardized values demonstrated hierarchical clustering of gut microbial taxa with anthropometric and metabolic variables (Figure 3). SCFA-producing taxa, including *Faecalibacterium*, *Akkermansia*, *Ruminococcus*, and *Lachnospiraceae*, clustered together and showed inverse associations with central adiposity, triglyceride concentrations, and systolic blood pressure, mirroring the observed negative correlations of *Ruminococcus* and *Lachnospiraceae* with glycemic and lipid parameters. In contrast, taxa positively associated with lipid and glycemic markers in the correlation analyses, such as *Prevotella*, *Eubacterium*, and *Bacteroides*, clustered with higher cardiometabolic risk parameters. Participants with metabolic syndrome grouped predominantly within this cluster, characterized by lower relative abundances of metabolically beneficial, SCFA-producing bacteria.

**Figure 4:**
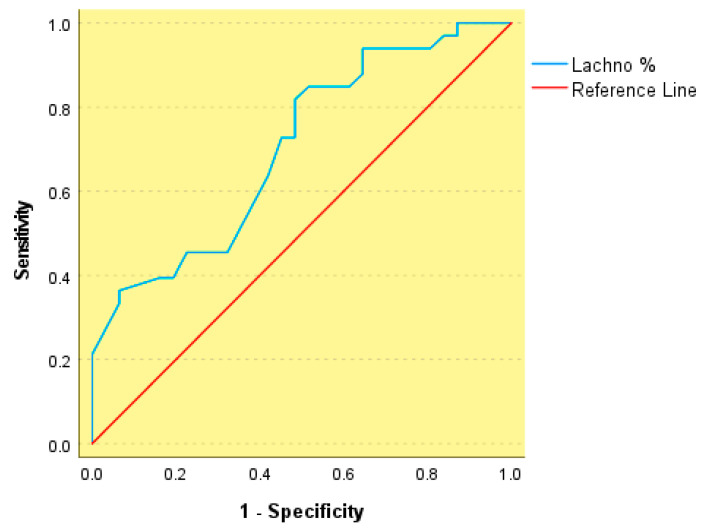
Receiver operating characteristic (ROC) curve of *Lachnospiraceae* relative abundance (Lachno %) for discrimination of metabolic syndrome.

Collectively, receiver operating characteristic (ROC) curve analyses identified selected gut microbiome-related bacterial parameters with statistically significant but moderate discriminative capacity for specific cardiometabolic conditions. Reduced relative abundance of *Lachnospiraceae* (Lachno %) was associated with metabolic syndrome, yielding an AUC of 0.702 (*p* = 0.002) and an optimal cutoff value of ≤18.0%, with a sensitivity of 82% and a specificity of 52% (Figure 4). Although statistically significant, this level of discrimination corresponds to poor to fair accuracy and does not support the use of *Lachnospiraceae* abundance as a standalone clinical classifier. Accordingly, these ROC-derived cutoffs should be interpreted as exploratory, post hoc discriminative thresholds within the studied cohort, rather than as clinically actionable or predictive biomarkers.

**Figure 5:**
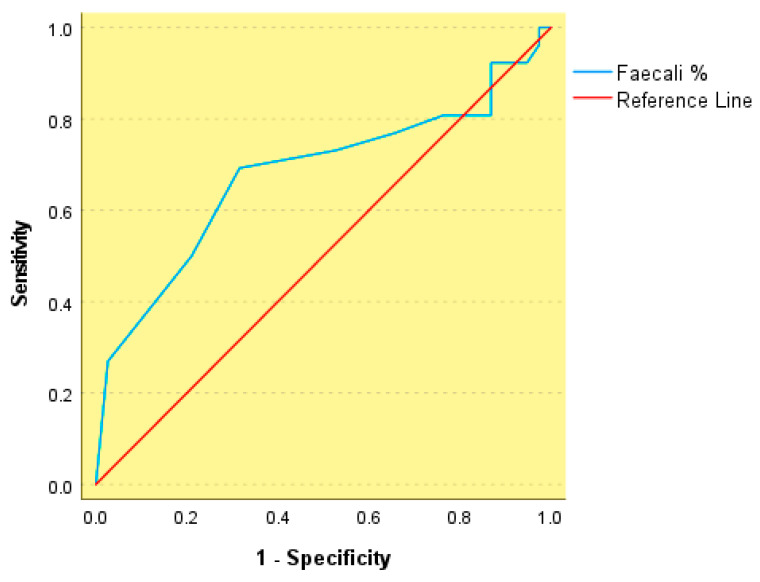
Receiver operating characteristic (ROC) curve of abundance *Faecalibacterium* relative abundance (Faecali %) for discrimination of metabolic syndrome.

In a similar manner, lower relative abundance of *Faecalibacterium* (Faecali %) was associated with arterial hypertension, yielding an AUC of 0.678 (*p* = 0.016) and an optimal cutoff value of ≤2.50%, with a sensitivity of 69% and a specificity of 68% (Figure 5). As with other ROC analyses, this level of discrimination reflects moderate to poor accuracy and does not support the use of *Faecalibacterium* abundance as a standalone clinical classifier. Accordingly, the identified cutoff should be interpreted as an exploratory, cohort-specific discriminative threshold rather than as a clinically actionable or predictive biomarker.

**Figure 6:**
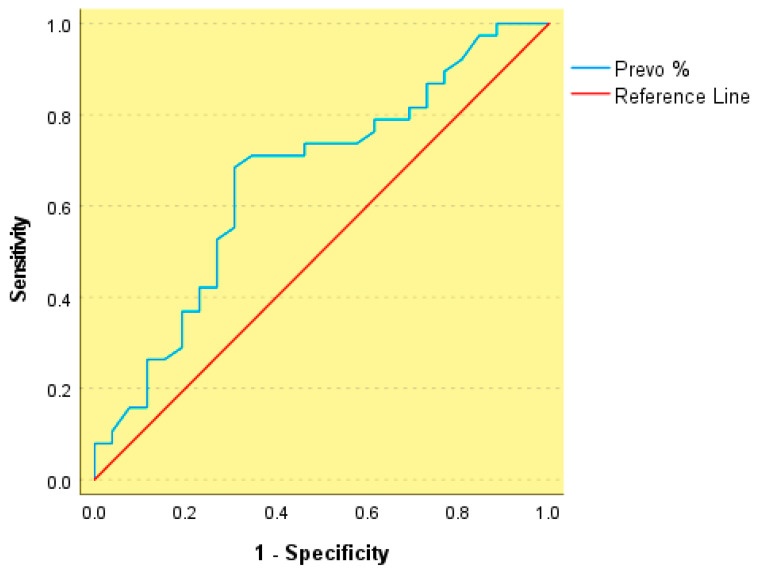
Receiver operating characteristic (ROC) curve of Prevotella relative abundance (Prevo %) for discrimination of metabolic syndrome.

Furthermore, higher relative abundance of *Prevotella* (Prevo %) was associated with dyslipidemia, yielding an AUC of 0.658 (*p* = 0.025) and an optimal threshold of ≥23.0%, with a sensitivity of 68% and a specificity of 69% (Figure 6). This degree of discrimination corresponds to poor to fair accuracy and does not support the use of *Prevotella* abundance as a standalone clinical classifier. Accordingly, the identified threshold should be interpreted as an exploratory, cohort-specific discriminative cutoff rather than as a clinically actionable or predictive biomarker.

Although the diagnostic performance of these microbiome-related bacterial markers was moderate, each demonstrated a statistically significant, cohort-specific discriminative threshold, indicating condition-specific, taxon-level gut microbial signatures within the studied population with obesity.

## 4. Discussion

Obesity is increasingly recognized as a biologically heterogeneous condition, characterized by marked interindividual variability in susceptibility to cardiometabolic complications such as metabolic syndrome, arterial hypertension, and dyslipidemia [17,18,19]. Within this context, alterations in selected gut bacterial taxa have been proposed as potential modifiers of metabolic homeostasis through interactions with host energy balance, immune regulation, intestinal barrier integrity, bile acid metabolism, and inflammatory signaling [6,19,20,21,22,23]. Although mechanistic pathways involving short-chain fatty acids have been extensively discussed in the literature, these mechanisms are inferred from prior experimental and clinical studies and were not directly assessed in the present work [21,22,23].

Despite the growing body of microbiome-related research in obesity, clinical translation has remained limited. Many studies rely on descriptive comparisons of microbial abundance or diversity metrics and rarely evaluate whether specific bacterial features demonstrate independent associations or quantifiable discriminative capacity [24,25,26,27]. Consequently, it remains unclear which microbial alterations represent epiphenomena of obesity and which are meaningfully linked to cardiometabolic risk. In this regard, the present study contributes by integrating correlation analyses, ROC-based discrimination, and multivariable modeling in a well-characterized cohort of individuals with obesity.

The present findings indicate that cardiometabolic risk in obesity is not associated with generalized dysbiosis but rather with selective, functionally coherent alterations in specific bacterial taxa. Only a limited subset of microbiome-related parameters demonstrated consistent associations with metabolic syndrome and its components, underscoring the importance of targeted, taxon-specific interpretation. Simplified dysbiosis indices, such as bacterial ratios, did not differ meaningfully between groups and showed no relevant discriminative performance, supporting concerns regarding the biological and clinical limitations of reductive microbial markers that fail to capture functional complexity and host–environment interactions [24,25].

Among the evaluated taxa, reduced relative abundance of *Lachnospiraceae* emerged as the most robust microbial feature associated with metabolic syndrome. Although the identified ROC-derived cutoff demonstrated only moderate discriminative ability, the association remained independent of age and sex in multivariable analyses. Importantly, AUC values in the range of 0.65–0.70 are generally considered to reflect poor to fair discrimination and do not support the use of individual bacterial taxa as standalone clinical biomarkers. Rather, these findings suggest a complementary role within broader cardiometabolic risk assessment frameworks. The inverse associations observed between *Lachnospiraceae* abundance and central adiposity, fasting glucose, and insulin resistance align with prior reports linking depletion of butyrate-producing taxa to metabolic vulnerability [12,26,27,28,29]. Nevertheless, mechanistic interpretations remain inferential, as SCFA concentrations were not measured in the present study.

A similar pattern was observed for *Faecalibacterium*, whose reduced relative abundance was associated with arterial hypertension. *Faecalibacterium prausnitzii* is widely described as an anti-inflammatory commensal with a central role in SCFA production, and its depletion has been reported in cardiometabolic and cardiovascular disorders [30,31,32]. Experimental evidence suggests that SCFA-producing bacteria may influence vascular tone and blood pressure regulation through multiple pathways, including modulation of G-protein-coupled receptors, sympathetic activity, and the renin–angiotensin–aldosterone system [33,34,35,36,37,38]. However, such mechanisms were not directly examined in the present study, and the observed associations should therefore be interpreted as reflective of potential links rather than causal effects.

In contrast, increased *Prevotella* abundance was associated with dyslipidemia and adverse lipid profiles. The metabolic implications of *Prevotella* enrichment appear to be context-dependent, with beneficial associations reported in high-fiber dietary settings and unfavorable effects observed in Westernized nutritional environments [39,40,41]. In the present cohort, positive correlations between *Prevotella* abundance and LDL cholesterol, triglycerides, and total cholesterol are consistent with observations linking *Prevotella*-dominant configurations to altered bile acid metabolism, immune activation, and lipid dysregulation [41,42]. These findings further emphasize that microbial effects cannot be interpreted independently of host and environmental context.

Exploratory clustering analyses provided additional support for the taxon-specific nature of these associations. A configuration enriched in SCFA-producing taxa, including *Faecalibacterium*, *Akkermansia*, *Ruminococcus*, and members of the Lachnospiraceae family, aligned with more favorable metabolic profiles, whereas a configuration dominated by *Prevotella* and other taxa associated with lipid abnormalities clustered with metabolic syndrome features [24,25]. Importantly, this patterning reflects associations among a predefined set of bacterial taxa assessed by targeted RT-qPCR and does not represent comprehensive gut microbiome ecosystem profiling.

Several limitations warrant consideration. The cross-sectional design precludes causal inference, and microbiome-related analyses were based on a single stool sample per participant without assessment of temporal variability [28]. Smoking prevalence differed markedly between groups and represents a potential environmental confounder influencing both cardiometabolic outcomes and gut bacterial composition [4,16]. Although dietary intake was assessed, dietary variables were not incorporated into multivariable models due to variability and sample size constraints. In addition, while sex was included as an adjustment variable, the study was not powered to evaluate sex-specific microbiome–host interactions reported in the recent literature [42].

Taken together, the present findings suggest that cardiometabolic risk in obesity is associated with selective alterations in specific gut bacterial taxa rather than global microbial disruption. These associations are exploratory and hypothesis-generating and highlight the potential value of targeted microbial markers as complementary indicators of metabolic vulnerability. Future longitudinal studies incorporating comprehensive microbiome profiling and direct metabolite measurements are required to clarify causality and clinical relevance [17,19,28].

## 5. Study Limitations

Several limitations should be acknowledged. First, gut microbiome assessment was performed using a targeted qPCR-based approach, which focuses on a predefined set of functionally relevant bacterial taxa and does not capture overall microbial diversity, strain-level variation, or comprehensive functional capacity. Second, the cross-sectional design precludes causal inference. Although ROC analyses identified statistically significant discriminative thresholds, these values represent cohort-specific, exploratory cutoffs rather than validated predictors of cardiometabolic risk. Third, although sex was included as an adjustment variable in multivariable models, the study was not powered to perform robust sex-stratified or interaction analyses. Finally, smoking prevalence differed substantially between groups and may represent an important confounder influencing both cardiometabolic outcomes and gut microbiome composition.

## 6. Conclusions

In conclusion, cardiometabolic risk in obesity is associated with selective, taxon-specific gut microbiome signatures rather than generalized dysbiosis. Reduced *Lachnospiraceae* abundance and lower levels of other SCFA-producing bacteria were associated with adverse metabolic phenotypes, while increased *Prevotella* abundance characterized dyslipidemia-related profiles. These findings are exploratory and hypothesis-generating and warrant confirmation in longitudinal studies and independent population.

## Figures and Tables

**Table 1:** Baseline anthropometric characteristics of the study groups.

Variable	Group 1: Obesity (*n* = 50)	Group 2: MS (*n* = 50)	*p* Value
Age (years)	43.74 ± 11.75	48.42 ± 12.66	0.148
BMI (kg/m^2^)	34.05 ± 4.02	33.74 ± 3.09	0.629
Waist circumference (cm)	96.77 ± 11.69	106.44 ± 11.51	<0.001
Waist-to-hip ratio (WHR)	0.85 [0.78–0.88]	0.92 [0.84–0.99]	<0.001
Waist-to-stature ratio (WSR)	0.49 ± 0.03	0.66 ± 0.07	0.008
Body fat (%)	93.50 [85.00–98.25]	106.00 [100.00–115.30]	<0.001
Visceral fat rating (VFR)	8.84 ± 2.88	11.21 ± 3.73	0.009

Note. MS—metabolic syndrome; BMI—body mass index; WHR—waist-to-hip ratio; WSR—waist-to-stature ratio; VFR—visceral fat ration. Data are presented as mean ± standard deviation (SD) for normally distributed variables and as median [interquartile range (IQR25–IQR75)] for non-normally distributed variables. *p*-values were calculated using Student’s *t*-test or Mann–Whitney *U* test, as appropriate. A two-sided *p* < 0.05 was considered statistically significant.

**Table 2:** Cardiovascular risk factors in the study groups.

Parameter	Group 1: Obesity (*n* = 50)	Group 2: MS (*n* = 50)	*p*-Value
SBP (mmHg)	120 [110–130]	140 [130–152.5]	0.023
DBP (mmHg)	80 [80–90]	80 [80–90]	ns
Total cholesterol (mmol/L)	5.3 [4.5–5.5]	6.2 [4.4–7.3]	0.042
LDL-C (mmol/L)	3.21 ± 1.01	3.62 ± 0.86	ns
HDL-C (mmol/L)	1.32 ± 0.27	1.21 ± 0.28	ns
Triglycerides (mmol/L)	1.46 ± 0.69	2.17 ± 0.95	0.007
Hypertension (%)	33.3	77.4	0.006
Smoking (%)	26.7	73.1	<0.001
Dyslipidemia (%)	31	74.2	0.002

Note. Data are presented as mean ± standard deviation (SD) for normally distributed variables and as median [interquartile range (IQR25–IQR75)] for non-normally distributed variables. *p*-values were calculated using *Student’s t-test or Mann–Whitney U test*, as appropriate. Categorical variables were compared using the *χ*^2^ *test* or *Fisher’s* exact test. *ns* indicates a non-significant difference. SBP—systolic blood pressure; DBP—diastolic blood pressure; LDL-C—low-density lipoprotein cholesterol; HDL-C—high-density lipoprotein cholesterol.

## Data Availability

The data presented in this study are available upon request from the corresponding authors due to patient privacy.

## References

[1] Loos R.J.F., Yeo G.S.H. (2022). The Genetics of Obesity: From Discovery to Biology. Nat. Rev. Genet..

[2] Hall K.D., Heymsfield S.B., Kemnitz J.W., Klein S., Schoeller D.A., Speakman J.R. (2012). Energy balance and its components: Implications for body weight regulation. Am. J. Clin. Nutr..

[3] Saltiel A.R., Olefsky J.M. (2017). Inflammatory Mechanisms Linking Obesity and Metabolic Disease. J. Clin. Investig..

[4] Fan Y., Pedersen O. (2021). Gut Microbiota in Human Metabolic Health and Disease. Nat. Rev. Microbiol..

[5] Aron-Wisnewsky J., Clément K., Nieuwdorp M. (2019). Fecal Microbiota Transplantation: A Future Therapeutic Option for Obesity/Diabetes?. Curr. Diab. Rep..

[6] Crovesy L., Masterson D., Rosado E.L. (2020). Profile of the gut microbiota of adults with obesity: A systematic review. Eur. J. Clin. Nutr..

[7] Sonnenburg J.L., Bäckhed F. (2016). Diet–Microbiota Interactions as Moderators of Human Metabolism. Nature.

[8] Gurung M., Li Z., You H., Rodrigues R., Jump D.B., Morgun A., Shulzhenko N. (2020). Role of Gut Microbiota in Type 2 Diabetes Pathophysiology. EBioMedicine.

[9] Liu B.N., Liu X.T., Liang Z.H., Wang J.H. (2021). Gut Microbiota in Obesity. World J. Gastroenterol..

[10] Canfora E.E., Meex R.C.R., Venema K., Blaak E.E. (2019). Gut Microbial Metabolites in Obesity, NAFLD and T2DM. Nat. Rev. Endocrinol..

[11] Dalile B., Van Oudenhove L., Vervliet B., Verbeke K. (2019). The Role of Short-Chain Fatty Acids in Microbiota–Gut–Brain Communication. Nat. Rev. Gastroenterol. Hepatol..

[12] Cani P.D., Amar J., Iglesias M.A., Poggi M., Knauf C., Bastelica D., Neyrinck A.M., Fava F., Tuohy K.M., Chabo C. (2007). Metabolic Endotoxemia Initiates Obesity and Insulin Resistance. Diabetes.

[13] Cani P.D. (2018). Human Gut Microbiome: Hopes, Threats and Promises. Gut.

[14] Fiorucci S., Distrutti E. (2015). Bile Acid-Activated Receptors, Intestinal Microbiota, and the Treatment of Metabolic Disorders. Trends Mol. Med..

[15] Drucker D.J. (2013). Incretin Action in the Pancreas: Potential Promise, Possible Perils, and Pathological Pitfalls. Diabetes.

[16] Cryan J.F., O’Riordan K.J., Cowan C.S.M., Sandhu K.V., Bastiaanssen T.F.S., Boehme M., Codagnone M.G., Cussotto S., Fulling C., Golubeva A.V. (2019). The Microbiota–Gut–Brain Axis. Physiol. Rev..

[17] Gérard P. (2016). Gut microbiota and obesity. Cell. Mol. Life Sci..

[18] Pedersen H.K., Gudmundsdottir V., Nielsen H.B., Hyotylainen T., Nielsen T., Jensen B.A.H., Forslund K., Hildebrand F., Prifti E., Falony G. (2016). Human gut microbes impact host serum metabolome and insulin sensitivity. Nature.

[19] Canfora E.E., Jocken J.W., Blaak E.E. (2015). Short-chain fatty acids in control of body weight and insulin sensitivity. Nat. Rev. Endocrinol..

[20] Koh A., De Vadder F., Kovatcheva-Datchary P., Bäckhed F. (2016). From dietary fiber to host physiology: Short-chain fatty acids as key bacterial metabolites. Cell.

[21] Le Chatelier E., Nielsen T., Qin J., Prifti E., Hildebrand F., Falony G., Almeida M., Arumugam M., Batto J.-M., Kennedy S. (2013). Richness of human gut microbiome correlates with metabolic markers. Nature.

[22] Karlsson F.H., Tremaroli V., Nookaew I., Bergström G., Behre C.J., Fagerberg B., Nielsen J., Bäckhed F. (2013). Gut metagenome in European women with normal, impaired and diabetic glucose control. Nature.

[23] Liu R., Hong J., Xu X., Feng Q., Zhang D., Gu Y., Shi J., Zhao S., Liu W., Wang X. (2017). Gut microbiome and serum metabolome alterations in obesity and after weight-loss intervention. Nat. Med..

[24] Qin J., Li Y., Cai Z., Li S., Zhu J., Zhang F., Liang S., Zhang W., Guan Y., Shen D. (2012). A metagenome-wide association study of gut microbiota in type 2 diabetes. Nature.

[25] Allin K.H., Nielsen T., Pedersen O. (2015). Mechanisms in endocrinology: Gut microbiota in patients with type 2 diabetes mellitus. Eur. J. Endocrinol..

[26] Louis P., Flint H.J. (2009). Diversity, metabolism and microbial ecology of butyrate-producing bacteria from the human large intestine. FEMS Microbiol. Lett..

[27] den Besten G., van Eunen K., Groen A.K., Venema K., Reijngoud D.-J., Bakker B.M. (2013). The role of short-chain fatty acids in the interplay between diet, gut microbiota, and host energy metabolism. J. Lipid Res..

[28] Chambers E.S., Preston T., Frost G., Morrison D.J. (2018). Role of Gut Microbiota-Generated Short-Chain Fatty Acids in Metabolic and Cardiovascular Health. Curr. Nutr. Rep..

[29] Sokol H., Pigneur B., Watterlot L., Lakhdari O., Bermúdez-Humarán L.G., Gratadoux J.-J., Blugeon S., Bridonneau C., Furet J.-P., Corthier G. (2008). *Faecalibacterium prausnitzii* is an anti-inflammatory commensal bacterium identified by gut microbiota analysis of Crohn disease patients. Proc. Natl. Acad. Sci. USA.

[30] Quévrain E., Maubert M.A., Michon C., Chain F., Marquant R., Tailhades J., Miquel S., Carlier L., Bermúdez-Humarán L.G., Pigneur B. (2016). Identification of an anti-inflammatory protein from *Faecalibacterium prausnitzii,* a commensal bacterium deficient in Crohn’s disease. Gut.

[31] Lopez-Siles M., Duncan S.H., Garcia-Gil L.J., Martinez-Medina M. (2017). *Faecalibacterium prausnitzii*: From microbiology to diagnostics and prognostics. ISME J..

[32] Yan Q., Gu Y., Li X., Yang W., Jia L., Chen C., Han X., Huang Y., Zhao L., Li P. (2017). Alterations of the Gut Microbiome in Hypertension. Front. Cell Infect. Microbiol..

[33] Li J., Zhao F., Wang Y., Chen J., Tao J., Tian G., Wu S., Liu W., Cui Q., Geng B. (2017). Gut microbiota dysbiosis contributes to the development of hypertension. Microbiome.

[34] Tang W.H.W., Kitai T., Hazen S.L. (2017). Gut microbiota in cardiovascular health and disease. Circ. Res..

[35] Pluznick J.L., Protzko R.J., Gevorgyan H., Peterlin Z., Sipos A., Han J., Brunet I., Wan L.-X., Rey F., Wang T. (2013). Olfactory receptor responding to gut microbiota-derived signals plays a role in renin secretion and blood pressure regulation. Proc. Natl. Acad. Sci. USA.

[36] Natarajan N., Hori D., Flavahan S., Steppan J., Flavahan N.A., Berkowitz D.E., Pluznick J.L. (2016). Microbial short chain fatty acid metabolites lower blood pressure via endothelial G protein-coupled receptor 41. Physiol. Genom..

[37] Marques F.Z., Nelson E., Chu P.-Y., Horlock D., Fiedler A., Ziemann M., Tan J.K., Kuruppu S., Rajapakse N.W., El-Osta A. (2017). High-fiber diet and acetate supplementation change the gut microbiota and prevent the development of hypertension and heart failure in hypertensive mice. Circulation.

[38] Tett A., Pasolli E., Masetti G., Ercolini D., Segata N. (2021). Prevotella diversity, niches and interactions with the human host. Nat. Rev. Microbiol..

[39] Wu G.D., Chen J., Hoffmann C., Bittinger K., Chen Y.-Y., Keilbaugh S.A., Bewtra M., Knights D., Walters W.A., Knight R. (2011). Linking long-term dietary patterns with gut microbial enterotypes. Science.

[40] Larsen J.M. (2017). The immune response to Prevotella bacteria in chronic inflammatory disease. Immunology.

[41] Caesar R., Tremaroli V., Kovatcheva-Datchary P., Cani P.D., Bäckhed F. (2015). Crosstalk between gut microbiota and dietary lipids aggravates WAT inflammation through TLR signaling. Cell Metab..

[42] Wahlström A., Sayin S.I., Marschall H.-U., Bäckhed F. (2016). Intestinal crosstalk between bile acids and microbiota and Its Impact on Host Metabolism. Cell Metab..

